# Gene-Wise Association of Variants in Four Lysosomal Storage Disorder Genes in Neuropathologically Confirmed Lewy Body Disease

**DOI:** 10.1371/journal.pone.0125204

**Published:** 2015-05-01

**Authors:** Lorraine N. Clark, Robin Chan, Rong Cheng, Xinmin Liu, Naeun Park, Nancy Parmalee, Sergey Kisselev, Etty Cortes, Paola A. Torres, Gregory M. Pastores, Jean P. Vonsattel, Roy Alcalay, Karen Marder, Lawrence L. Honig, Stanley Fahn, Richard Mayeux, Michael Shelanski, Gilbert Di Paolo, Joseph H. Lee

**Affiliations:** 1 Taub Institute for Research on Alzheimer’s Disease and the Aging Brain, Columbia University, New York, New York, United States of America; 2 Department of Pathology and Cell Biology, Columbia University, New York, New York, United States of America; 3 Gertrude H. Sergievsky Center, Columbia University, New York, New York, United States of America; 4 Department of Neurology, New York University School of Medicine, New York, New York, United States of America; 5 Department of Pediatrics, New York University School of Medicine, New York, New York, United States of America; 6 Department of Neurology, Columbia University, New York, New York, United States of America; 7 Department of Psychiatry, and Department of Statistics, Columbia University, New York, New York, United States of America; 8 Department of Epidemiology, Mailman School of Public Health, Columbia University, New York, New York, United States of America; 9 Center for Human Genetics, Columbia University, New York, New York, United States of America; Icahn School of Medicine at Mount Sinai, UNITED STATES

## Abstract

**Objective:**

Variants in *GBA* are associated with Lewy Body (LB) pathology. We investigated whether variants in other lysosomal storage disorder (LSD) genes also contribute to disease pathogenesis.

**Methods:**

We performed a genetic analysis of four LSD genes including *GBA*, *HEXA*, *SMPD1*, and *MCOLN1* in 231 brain autopsies. Brain autopsies included neuropathologically defined LBD without Alzheimer Disease (AD) changes (n = 59), AD without significant LB pathology (n = 71), Alzheimer disease and lewy body variant (ADLBV) (n = 68), and control brains without LB or AD neuropathology (n = 33). Sequencing of *HEXA*, *SMPD1*, *MCOLN1* and *GBA* followed by ‘gene wise’ genetic association analysis was performed. To determine the functional effect, a biochemical analysis of *GBA* in a subset of brains was also performed. GCase activity was measured in a subset of brain samples (n = 64) that included LBD brains, with or without *GBA* mutations, and control brains. A lipidomic analysis was also performed in brain autopsies (n = 67) which included LBD (n = 34), ADLBV (n = 3), AD (n = 4), PD (n = 9) and control brains (n = 17), comparing GBA mutation carriers to non-carriers.

**Results:**

In a ‘gene-wise’ analysis, variants in *GBA*, *SMPD1* and *MCOLN1* were significantly associated with LB pathology (*p* range: 0.03–4.14 x10^-5^). Overall, the mean levels of GCase activity were significantly lower in *GBA* mutation carriers compared to non-carriers (*p*<0.001). A significant increase and accumulation of several species for the lipid classes, ceramides and sphingolipids, was observed in LBD brains carrying *GBA* mutations compared to controls (*p* range: *p*<0.05-*p*<0.01).

**Interpretation:**

Our study indicates that variants in *GBA*, *SMPD1* and *MCOLN1* are associated with LB pathology. Biochemical data comparing *GBA* mutation carrier to non-carriers support these findings, which have important implications for biomarker development and therapeutic strategies.

## Introduction

Lewy body disorders (LBD) which include Parkinson’s Disease (PD) and Dementia with Lewy bodies (DLB) are characterized by neuronal loss in the substantia nigra (SN) and the presence of neuronal cytoplasmic inclusions composed predominantly of α-synuclein termed Lewy Bodies (LBs)[1–3]. α-synuclein immunoreactivity, including LB, have been described as features seen in the neuropathology of several lysosomal storage disorders including notably Gaucher disease (GD), but also Sandhoff disease, Tay Sachs disease, and Sanfilippo syndrome[4–8]. Heterozygosity for mutations in the gene encoding glucocerebrosidase (*GBA*), which cause Gaucher disease (GD), has been identified as a risk factor for both PD and DLB. We and others have shown that in sporadic and familial PD, *GBA* mutations are associated with early-onset PD and may modify age at onset of PD[9,10] and that in brain autopsies *GBA* mutation status was significantly associated with the presence of cortical LB (OR = 6.48, 95% CI, 2.45–17.16, p<0.001) and a neuropathological diagnosis of DLB after adjusting for sex, age at death, and presence of APOE-4[11]. A recent study that assessed the association of specific founder mutations in each of the lysosomal storage disorder genes *HEXA*, *SMPD1* and *MCOLN1*, in 938 Ashkenazi Jewish (AJ) PD patients and 282 matched AJ controls, reported *SMPD1* L302P as a risk factor for PD in the AJ population[12].

To determine whether variants in other lysosomal storage disease genes, in the same pathway as *GBA*, are associated with LBs we conducted an independent genetic study of the lysosomal storage disorder genes *GBA*, *HEXA*, *SMPD1*, and *MCOLN1* in 231 brain autopsies from the New York Brain Bank at Columbia University. Brain autopsies included neuropathologically defined LBD without AD changes (n = 59), AD without significant LB pathology (n = 71), ADLBV (n = 68), and control brains without LB or AD neuropathology (n = 33). The functional effect of GBA mutations was also determined by performing a biochemical analysis of *GBA* in a subset of brains.

## Materials and Methods

### Clinical material

Brain tissue samples were obtained from the New York Brain Bank at Columbia University including cases obtained through the Alzheimer’s Disease Research Center and the Center for Parkinson’s Disease and Other Movement Disorders. Brain autopsies included neuropathologically defined LBD without AD changes (n = 59), AD without significant LB pathology (n = 71), ADLBV (n = 68), and control brains without LB or AD neuropathology (n = 33). (Table 1 and S1 Table). LB and Alzheimer plaque and tangle pathology was assessed according to published guidelines as described previously[13–15] (see S1 Methods for a detailed description of neuropathological evaluation). Clinical information on dementia was available for 208 brain autopsy samples (see S1 Methods). While the primary analysis in this paper is of autopsy proven cases, an additional group of living controls (128 Ashkenazi Jewish (AJ) healthy individuals were used in a secondary analysis to supplement the limited number of brain autopsy controls (see S1 Methods for a description of the controls). Columbia University Institutional Review Board approved the protocols and consent procedures. Written informed consent was obtained from all participants in the study.

**Table 1:** Characteristics of Autopsy Subjects.

All autopsies		LBD	ADLBV	AD	Control	Total
** **	**N**	59	68	71	33	231
**Male**	**%**	71.2	52.9	36.6	54.5	52.8
**Age at Dementia (yr) **	**Mean**	66.6	67.6	70.7		68.4
	**SD**	10.3	9.6	8.5		9.5
**Age at Death (yr)**	**Mean**	78.3	79.1	81.6	70.3	78.4
	**SD**	8.7	8.5	8.6	14.2	10.1
**Duration (yr)**	**Mean**	11.6	10.3	10.0		10.6
	**SD**	6.1	6.1	4.3		5.5
**Education (yr) **	**Mean**	16.6	14.1	13.8	14.2	14.5
	**SD**	2.3	4.1	4.3	3.4	4.0
**Ethnicity**	**% White**	94.9	86.8	85.9	60.6	84.8
**%AJ ancestry**	**N (individuals)**	3	7	15	2	27
	**% AJ (n/total samples with GWAS)**	100(3/3)	35 (7/20)	41.67 (15/36)	66.67 (2/3)	43.5** (27/62)
**LB Pathology Present**	**%**	100.0	100.0	16.9	3.0	60.6
**LB Cortical Pathology Present **	**%**	100.0	100.0	0.0	0.0	55.0
**LB Subcortical Pathology Present**	**%**	64.4	69.1	12.7	0.0	40.7
**AD Pathology Present**	**%**	79.7	100.0	100.0	33.3	85.3
**AD Pathological Diagnosis**	**%**	0.0	100.0	100.0	0.0	60.2
**GBA mutation**	**N(Individuals)**	28	16	6	1	51
	**%**	47.5	23.5	8.5	3.0	22.1
**SMPD1 mutation**	**N(Individuals)**	12	14	4	3	33
	**%**	20.3	20.6	5.6	9.1	14.3
**HEXA mutation**	**N(Individuals)**	8	17	8	6	39
	**%**	13.6	25.0	11.3	18.2	16.9
**MCOLN1 mutation **	**N(Individuals)**	17	20	17	8	62
	**%**	28.8	29.4	23.9	24.2	26.8
APOE (no E4)*	**N(Individuals)**	33	26	26	24	109
	**%**	66.0	41.9	42.6	77.4	47.2
APOE (one E4) *	**N(Individuals)**	13	26	25	6	70
	**%**	26.0	41.9	41.0	19.4	30.3
APOE (two E4) *	**N(Individuals)**	4	10	10	1	25
	**%**	8.0	16.1	16.4	3.2	10.8

* APOE missing in 27cases

**%AJ in brain autopsy sample with GWAS data available (n = 62)

### Population Stratification and Ashkenazi Jewish Ancestry

Since a founder effect for LSD gene mutations have been reported in the AJ population we also determined AJ ancestry in brain autopsy samples. Information about AJ ancestry was not available for brain autopsies. We used two methods to examine AJ ancestry and underlying population structure in brain autopsies. In the first method, Multidimensional scaling (MDS) as implemented in the program PLINK (Version 1.07) for detecting population outliers and adjusting for population stratification was used. Briefly, we used 288, 963 autosomal SNPs for brain autopsies (n = 62), augmented with 252 AJ samples with subjects from the HapMap website (http:www.hapmap.org/), which included 90 CEU, 90 Yorubans and 90 Asians. The best fitting model assumed two underlying populations with overlap of 27 white brain autopsies with the AJ cluster and the remainder of the white brain autopsies with the white CEU cluster. In the second method, principle component analysis (PCA) as implemented in the GCTA package [16] was used to examine ancestry and admixture in white brain autopsies, AJ samples together with subjects from HapMap. Projection of all the sample genotypes along the two principle components (PC2 and PC3) is shown in S1 Methods. As in the MDS analysis performed in PLINK, there is tight clustering of 27 brain autopsies with AJ sample cluster and the remainder of the white brain autopsies cluster with CEU samples.

### Molecular Genetic Analysis

Frozen cerebellar tissue was used to extract DNA. Sequencing of all *GBA* exons was performed as described previously[9]. Sequencing of all exons of *HEXA*, *SMPD1* and *MCOLN1* was also performed. Details of PCR and sequencing primers are available upon request. APOE genotyping was performed by MALDI-TOF mass spectrometry on the Sequenom platform as described previously[11].

### Analysis of functional effect of variants

The National Center for Biotechnology information (NCBI), ClinVar, the NHLBI Exome Sequencing project (ESP) exome variant server in addition to *in silico* prediction was used to assess the deleterious effect of variants.

### Enzyme Activity Measurements

For enzyme activity measurements, a subset (n = 64) of the total autopsy sample (n = 231) for which frozen brain tissue was available was selected based on neuropathological diagnosis and *GBA* mutation carrier status (*GBA* mutation carriers (n = 16), LBD brains without *GBA* mutations (n = 18) and control brains (n = 30)). Brain autopsy tissue (Cerebellum, BA4 and BA9 and ScxV) samples were homogenized in water (10% wt./vol.) using a Misonix Sonic Dismembrator and centrifuge at 30,000 Xg for 20 min. Protein concentration was determined using the Lowry method. The reaction mixture for β -glucocerebrosidase determination consisted of 50ug of protein, 50ul of 20mM 4-methylumbelliferyl-β-D-glucopyranoside, 10ul of 1M Citrate-Phosphate pH 5.0 and 10ul of 2% Sodium Tauro Deoxycholate. The reaction mixture was incubated at 37°C for 2Hours and then subsequently stopped with 2 ml 0.2 M glycine buffer, pH 10.3. The Hexosamindase A enzymatic reaction mixture consisted of 10ug of protein and 100ul of 3mM 4-methylumbelliferyl-2-acetoamido-2-deoxy-b-D-glucopyranoside in Citrate-Phosphate buffer pH4.0. Samples were incubated at 37°C for 10min and 0.2M glycine buffer was also used to stop the reaction. Fluorescence was determined in fluorescence spectrophotometer (Hitachi F-2500) at an excitation wavelength of 365 nm and emission wavelength of 448 nm. Samples were compared against a 4-methylumbelliferone (4-MU) standard curve prepared in 0.2 M glycine buffer. Enzyme activities were calculated in nmoles of 4-MU hydrolyzed/mg protein/hr. LBD brains did not carry variants in any of the other LSD genes analyzed. Frozen post-mortem interval (PMI) was available for all autopsy tissue and PMI did not appear to influence GCase activity.

### Lipid Profiling

For lipid profiling, a convenience subset (n = 67) of the total autopsy sample (n = 231) was selected based on neuropathological diagnosis and GBA mutation carrier status that included LBD brains from *GBA* mutation carriers (n = 13), LBD brains without *GBA* mutations (n = 33), AD brains (n = 4) and control brains (n = 17). Characteristics of the autopsy subjects are provided in S3 Table. Lipid extracts were prepared using a modified Bligh/Dyer extraction procedure, spiked with appropriate internal standards. The samples were analysed using an Agilent 1260 HPLC system coupled to an Agilent 6490 Triple Quadrupole mass spectrometer. The lipidomic profiles generated for each sample were obtained through a combination of HPLC separation and mass spectrometry in multiple reactions monitoring mode which allows for the unambiguous identification of lipids as described previously [17,18]. (S1 Methods).

### Statistical Analysis

T tests and chi square tests were used to compare continuous and categorical variables respectively. To determine whether a gene, represented by multiple sequenced variants, is associated with affection status or not, we applied the sequence kernal association test (SKAT) algorithm [19]. As above, age and sex were included in one model as covariates, and permutation based p-value was computed. To determine whether multiple variants in the lysosomal disease genes are associated with LB pathology in an additive manner, after correcting for age and sex as covariates, we applied multiple logistic regression. Although this additive model is simplistic, we reasoned that this is one way to gain insight into a set of functional (i.e., nonsynonymous) variants in the common disease pathway.

For lipidomics data, Statistical analysis for the AD and LBD mutation samples was based on the one way analysis of variance followed by post hoc Fisher’s least significant difference test while the LBD wild type samples was based on Student’s T-test. In all cases, *, **p** < 0.05; **, **p** <0.01; ***, **p** <0.001.

## Results

### Demographic and Neuropathological Characteristics of Autopsy Samples

The basic demographic and neuropathologic information of autopsy samples analysed is shown in Table 1 (all autopsies, N = 231) and S1 Table (white non-Hispanic ethnicity only, N = 196). Overall, the proportion of men (71.2%) was higher in the LBD and Alzheimer disease and lewy body variant (ADLBV) group, compared with that in the AD group (36.6%). Overall, LBD patients had a significantly earlier age at onset of dementia (66.58±10.28 years vs. 70.71±8.46; *p* = 0.05), earlier age at death (78.26±8.66 years vs. 81.64±8.60; *p* = 0.04), and had more years of education (16.61±2.33 years vs. 13.84±4.34; *p* = 0.02) compared to patients with AD. *APOE4* allele frequencies did not differ from reported population frequencies in non-AD groups.

### Sequencing and Association Analysis: Variants identified and predicted impact on function

Overall, we identified 51 (22.1%) subjects with a *GBA* variant, 39 (16.9%) with a *HEXA* variant, 33 (14.3%) with an *SMPD1* variant and 62 (26.8%) with an *MCOLN1* variant (Table 1). Many of the variants that we identified have been reported previously as pathogenic mutations in patients with the associated lysosomal storage disorder (Table 2). LSD variants that were significantly associated in brain autopsies with a neuropathological diagnosis of LBD are shown in Table 2.

**Table 2:** Variants identified in brain autopsy samples.

Gene	Chr, genomic coordinates*	Protein (allele name)	dbSNP	MAF (1000 genomes)	Clinical significance**
GBA	1:155235002	p.R535H (p.R496H)	rs80356773	NA (rare)	**Pathogenic[20]**
	1:155235196	p.R502C (p.R463C)	rs80356771	NA (rare)	**Pathogenic[20]**
	1: 155235252	p.L483P (p.L444P)	rs421016	0.0034	**Pathogenic[20]**
	1:155235727	p.D448H (D409H)	rs1064651	NA (rare)	**Pathogenic[20]**
	1:155235843	p.N409S (N370S)	rs76763715	0.0006	**Pathogenic[20]**
	1:155236246	p.T408M (T369M)	rs75548401	0.0018	Uncertain significance
	1:155236376	p.E365L (p.E326K)	rs2230288	0.0050	**Pathogenic[20]**
	1:155237458	p.H294Q (p.H255Q)	rs367968666	NA (rare)	**Pathogenic[20]**
	1:155238228	p.W223R (p.W184R)	rs61748906	NA (rare)	**Pathogenic[20]**
	1:155238392	-	rs114099990	NA (rare)	Unknown
	1:155240660–155240661	p.Leu29AlafsX188 (84GG)	rs387906315	NA (rare)	**Pathogenic[20]**
	1:155236304	p.E388K	-	NA (rare)	Unknown
SMPD1	11:6390654	p.Q19R	rs144465428	NA (rare)	unknown
	11:6390705	p.V36A	rs1050228	0.4387	Benign/likely benign
	11:6390741–6390742	p.Leu49_Ser50insAL p.Leu49_Ser50insALAL	rs71056748	NA (rare)	Unknown
	11:6391701	p.D212D	rs7951904	0.1282	Benign/likely benign
	11:6392137	p.E358K	-	NA (rare)	Unknown
	11:6394233	p.G508R	rs1050239	0.15	Benign/likely benign
	11:6394336	p.R542L	-	NA (rare)	Unknown
	11:6394652	-	rs8164	0.1484	Unknown
	11:6392136	p.A357A	rs72896268	0.0034	Benign/likely benign
	11:6391966	p.V301I	rs2723669	0.0032	Unknown
	11:6390697	p.M33I	rs142178073	0.0038	Unknown
	11:6394029	p.G492S	rs144873307	0.0014	**Likely Pathogenic[21]**
	11:6394261	p.E517V	rs142787001	0.0014	**Likely Pathogenic[22]**
	11:6333377	p.R418Q	-	NA (rare)	Unknown
HEXA	15:72347852	-	rs2302449	0.0759	Unknown
	15:72346579–72346580	p.Y427I (1277_1278insTATC)	rs387906309	NA (rare)	**Pathogenic**
	15:72349307	-	rs73440586	0.0721	Unknown
	15:72350564	p.V253V	rs117513345	0.0016	Unknown
	15:72375964	p.S3S	rs1800428	0.0441	Unknown
	15:72350584	p.R247W	rs121907970	0.0004	**Pathogenic**
	15:72351103	-	rs117160567	0.0144	Unknown
	15:72345619	-	rs2288259	NA (rare)	Unknown
	15:72346551	p.I436V	rs1800431	NA (rare)	Benign/likely benign
	15:72350518	p.G269S	rs121907954	NA (rare)	**Pathogenic**
	15:72351103	*c*.*672 +30 T>G*	rs117160567	0.0144	Unknown[23]
	15:72351231	p.V192I	-	NA (rare)	Unknown
	15:72355693	-	rs10220917	0.0875	Unknown
MCOLN1	19:7526723	-	rs45513896	0.0222	Unknown
	19:7527537	p.P197S	rs145706318	NA (rare)	Unknown
	19:7528162	p.T261M	rs73003348	0.0026	Unknown
	19:7528283	-	rs2305889	0.2821	Unknown
	19:7529124	p.C386C	rs139922988	0.0004	Unknown
	19:7533531	p.G528G	rs145386883	0.0006	Unknown
	19:7533693	-	rs686796	0.0122	Unknown
	19:7527954	p.S257R	rs113261161	0.0088	Unknown
	19:7528685	p.R322R	rs61736600	0.0375	Unknown
	19:7528703	p.N328N	rs612862	0.2556	Unknown
	19:7526768	p.A138V	rs142259322	0.0008	Unknown
	19:7529625	p.S424S	rs147754092	0.0012	Unknown

All mutations are described as recommended at www.hgvs.org/mutnomen

*Chr and genomic coordinates as based on assembly GRCh38 and genome build 106.

**Clinical Significance was assessed based on citations (published articles and URLs) documenting the clinical significance or based on pathogenic status reported in dbSNP or ClinVar

### Single Gene Wise Association: Multiple variants in *GBA*, *SMPD1* and *MCOLN1* are associated with a neuropathological diagnosis of LBD

SNP-set (Sequence) Kernal Association Test (SKAT) was used to evaluate association of variants in *GBA*, *HEXA*, *SMPD1* and *MCOLN1* (Table 3). When evaluating all variants, strongest association was observed for *GBA* variants in LBD (*p* = 2.95 x10^-5^) and ADLBV (*p* = 3.59 x10^-2^) (Table 3). Risk variants in *GBA*, *SMPD1* and *MCOLN1* were also significantly associated with LBD (*p* range = 0.03–4.14x10^-5^) and ADLBV (*p* range = 0.02–0.01) pathology but not AD (Table 3). We also evaluated association of protective variants and observed association of variants in *SMPD1* in LBD (*p* = 0.03) and ADLBV (*p* = 0.02), but not AD, and *MCOLN1* variants in LBD (p = 0.02), ADLBV (*p* = 0.005) but not AD (Table 3).

**Table 3:** Gene wise association SKAT analysis* in all samples.

Sample size Gene	LBD vs. CTRL (n = 59 vs. 33)		ADLBV vs. CTRL (n = 68 vs. 33)		AD vs. CTRL (n = 71 vs. 33)	
	P value	Marker (n)**	P value	Marker (n) **	P value	Marker (n) **
**All variants**						
**GBA**	2.95x10^-5^	11	3.59x10^-2^	7	0.363	4
**SMPD1**	0.114	12	0.259	12	0.347	9
**HEXA**	0.885	9	0.450	13	0.638	7
**MCOLN1**	3.25x10^-2^	6	8.31x10^-2^	11	0.368	9
**GBA+SMPD1**	2.89x10^-4^	23	3.94x10^-2^	19	0.563	13
**GBA+SMPD1+MCOLN1**	1.29x10^-3^	29	5.09x10^-2^	30	0.492	22
**Risk variants** ^**1**^						
**GBA**	4.14x-10^-5^	11	1.27x10^-2^	6	0.356	4
**SMPD1**	1.93x10^-2^	10	6.50x10^-2^	10	7.68x10^-2^	5
**HEXA**	0.124	5	0.105	10	0.254	3
**MCOLN1**	3.33x10^-2^	4	2.50x10^-2^	9	8.78x10^-2^	7
**GBA+SMPD1**	3.87x10^-5^	21	8.35x10^-3^	16	5.85x10^-2^	9
**GBA+SMPD1+MCOLN1**	1.11x10^-4^	25	4.35x10^-3^	25	3.39x10^-2^	16
**Protective variants** ^**1**^						
**GBA**	-^2^		1	1	-^2^	
**SMPD1**	2.80x10^-2^	2	1.68x10^-2^	2	0.152	4
**HEXA**	0.360	4	0.553	3	0.166	4
**MCOLN1**	2.18x10^-2^	2	4.89x10^-3^	2	8.24x10^-2^	2
**GBA+SMPD1**	2.76x10^-2^	2	0.152	3	0.152	4
**GBA+SMPD1+MCOLN1**	1.79x10^-3^	4	2.38x10^-3^	5	4.29x10^-2^	6

*Corrected for covariates.

** Indicates number of markers included in the test.

^1^ Risk variants are variants more frequent among cases than controls; whereas, variants are considered protective when they are more frequent in controls than cases.

^2^ No protective variants were observed.

The following secondary analyses of the same SKAT models was also performed: 1) SKAT analysis of LSD variants with MAF<5% in all samples (n = 231)(Table 4), 2) SKAT analysis of LSD variants with MAF<5% in ‘white’ subjects only (n = 196) (Table 5) and 3) SKAT analysis of LSD variants in all samples (n = 231) using a larger control group which included the brain autopsy controls (n = 33) and the AJ controls (n = 128) (S2 Table). When we restricted the analysis to variants with MAF<5% (Table 4), strongest association was observed for GBA variants in LBD (p = 1.37x10^-4^). Risk variants in *GBA*, *SMPD1* and *MCOLN1* remained significantly associated with LBD (*p* range = 0.04–1.77x10^-4^) and ADLBV (*p* range = 0.04–0.02) pathology but not AD (Table 4). When we restricted the analysis to whites only with variants with MAF<5% (Table 5) strongest association was observed for *GBA* variants in LBD (*p* = 0.0118). Risk variants in *SMPD1* in ADLBV were also significant (*p* = 0.0274). Although not significant, there was a trend towards significance for *MCOLN1* risk variants in LBD (*p* = 0.189) and ADLBV (*p* = 0.072). However, the small sample size of the ‘white’ controls (n = 20) in this stratified analysis may be a confounding factor and the results should be interpreted with caution.

**Table 4:** Gene wise SKAT analysis of LSD variants with MAF<5% in all samples.

Sample size Gene	LBD vs. CTRL (n = 59 vs. 33)		ADLBV vs. CTRL (n = 68 vs. 33)		AD vs. CTRL (n = 71 vs. 33)	
	P value	Marker (n)**	P value	Marker (n) **	P value	Marker (n) **
**All variants**						
**GBA**	1.37 x10^-4^	11	9.90x10^-2^	7	0.404	4
**SMPD1**	0.200	11	0.305	11	0.571	8
**HEXA**	0.663	7	0.470	10	0.233	5
**MCOLN1**	6.64x10^-2^	5	5.45x10^-2^	9	0.110	6
**GBA+SMPD1**	4.67 x10^-4^	22	8.18x10^-2^	18	0.644	12
**GBA+SMPD1+MCOLN1**	6.27 x10^-4^	27	2.94x10^-2^	27	0.344	18
**Risk variants** ^**1**^						
**GBA**	1.77x10^-4^	11	3.80x10^-2^	6	0.397	4
**SMPD1**	2.48x10^-2^	9	8.36x10^-2^	9	0.039	4
**HEXA**	6.82x10^-2^	4	6.27x10^-2^	8	0.522	2
**MCOLN1**	3.91x10^-2^	4	2.31x10^-2^	8	4.10x10^-2^	5
**GBA+SMPD1**	2.94x10^-5^	20	1.68x10^-2^	15	3.46x10^-2^	8
**GBA+SMPD1+MCOLN1**	9.07x10^-5^	24	7.60x10^-3^	23	1.78x10^-2^	13
**Protective variants** ^**1**^						
**GBA**	-^2^		0.824	1	-^2^	
**SMPD1**	0.253	2	5.73x10^-2^	2	0.615	4
**HEXA**	0.580	3	0.598	2	2.54x10^-2^	3
**MCOLN1**	0.373	1	0.810	1	0.448	1
**GBA+SMPD1**	0.253	2	0.262	3	0.615	4
**GBA+SMPD1+MCOLN1**	4.99x10^-2^	3	0.317	4	0.404	5

*Corrected for covariates.

** Indicates number of markers included in the test.

^1^ Risk variants are variants more frequent among cases than controls; whereas, variants are considered protective when they are more frequent in controls than cases.

^2^ No protective variants were observed.

**Table 5:** Gene wise SKAT analysis of LSD variants with MAF<0.05 in White subjects only.

Sample size Gene	LBD vs. CTRL (n = 56 vs. 20)		ADLBV vs. CTRL (n = 59 vs. 20)		AD vs. CTRL (n = 61 vs. 20)	
	P value	Marker (n) **	P value	Marker (n)**	P value	Marker (n) **
**All variants**						
**GBA**	1.18x10^-2^	10	0.306	7	0.795	4
**SMPD1**	0.702	9	0.443	10	0.693	7
**HEXA**	0.912	7	0.770	9	0.177	5
**MCOLN1**	0.194	4	0.129	8	0.117	5
**GBA+SMPD1**	3.54x10^-2^	19	0.205	17	0.853	11
**GBA+SMPD1+MCOLN1**	2.61x10^-2^	23	9.97x10^-2^	25	0.560	16
**Risk variants** ^**1**^						
**GBA**	1.02x10^-2^	10	0.126	6	0.543	3
**SMPD1**	0.276	8	0.027	7	0.149	4
**HEXA**	0.209	4	6.55x10^-2^	6	0.539	2
**MCOLN1**	0.189	4	7.19x10^-2^	7	5.86x10^-2^	4
**GBA+SMPD1**	7.30x10^-3^	18	6.60x10^-3^	13	0.113	7
**GBA+SMPD1+MCOLN1**	6.30x10^-3^	22	2.70x10^-3^	20	3.22x10^-2^	11
**Protective variants** ^**1**^						
**GBA**	-^2^		0.743	1	0.651	1
**SMPD1**	3.29x10^-2^	1	0.682	3	0.563	3
**HEXA**	0.517	3	0.606	3	1.93x10^-2^	3
**MCOLN1**	-^2^		0.794	1	0.826	1
**GBA+SMPD1**	3.29x10^-2^	1	0.772	4	0.432	4
**GBA+SMPD1+MCOLN1**	3.29x10^-2^	1	0.822	5	0.579	5

*Corrected for covariates.

** Indicates number of markers included in the test.

^1^ Risk variants are variants more frequent among cases than controls; whereas, variants are considered protective when they are more frequent in controls than cases.

SKAT analysis using the larger control group replicated the findings observed using the brain autopsy controls (n = 33) alone (S2 Table).

### Additive Effect of Multiple Variants in *GBA*, *SMPD1* and *MCOLN1*

In exploratory analyses logistic regression analysis was also used to determine whether multiple variants in the same disease pathway are associated with disease pathology in an additive manner after adjusting for age and sex as covariates. Strong associations (*p* range: 0.03–3.8x10^-5^) were also observed for LBD, ADLBV, and AD cases with multiple variants in *GBA*+*SMPD1* or *GBA*+*SMPD1*+*MCOLN1* (Table 3).

### GCase activity is decreased in LBD *GBA* mutation carriers compared to LBD non-carriers

**Fig 1:**
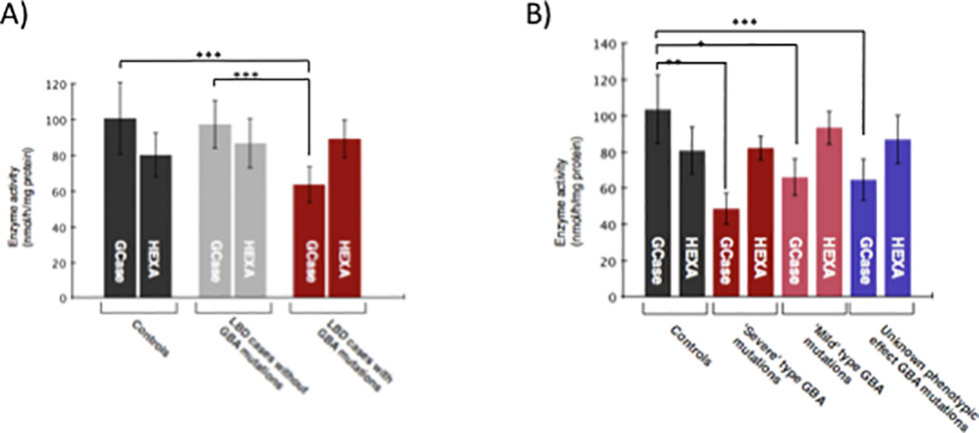
GCase and HexA activity in autopsy brain tissue. A) GCase activity was significantly reduced in LBD cases carrying *GBA* mutations (n = 16) compared to LBD non-*GBA* carriers (n = 18) and controls (n = 30). Differences in activity for *HEXA* were not significant in any group. B) GCase was significantly reduced in LBD cases with mutations classified as ‘severe’ type (L444P, 84insGG etc.) compared to controls, and to LBD cases with ‘mild’ mutations (N370S) or variants of unknown phenotypic effect (E326K, T369M). Differences in activity for *HEXA* were not significant. * *p*<0.05, ** *p*<0.01, *** *p*<0.001.

To determine whether carrier *GBA* mutation status was associated with reduced enzymatic activity (haploinsufficiency) we assayed GCase activity in a subset of autopsy samples (n = 64). GCase activity was measured in LBD brains from *GBA* mutation carriers (n = 16), LBD brains without *GBA* mutations (n = 18) and control brains (n = 30) (Fig 1) from the following brain regions Cerebellum, BA4 and BA9 and ScxV.

The enzyme activity of a second lysosomal hydrolase, α-hexosaminidase was also assayed to demonstrate specificity of decreased activity of GCase. Overall, the mean levels of GCase activity (*p*<0.001) and the β-glucocerebrosidase: α -hexosaminidase ratio (*p*<0.001) were significantly lower in *GBA* mutation carriers compared to non-carriers (Fig 1). We also observed significant differential enzyme activity of GCase or for the β-glucocerebrosidase:α-hexosaminidase ratio in subjects carrying *GBA* mutations classified phenotypically (as in Gaucher disease) as ‘severe’ type (e.g. 84insGG, L444P) (p<0.01) compared to subjects carrying *GBA* mutations classified phenotypically as ‘mild’ type (e.g. N370S, R496H) (p<0.05) or of unknown phenotypic effect (E326K, T369M) (p<0.001) compared to controls (Fig 1). Lastly, we examined the relation between GCase activity in subjects with a clinical diagnosis of dementia compared to cases without dementia. Overall, the mean levels of GCase activity (*p* = 0.0021) and the β-glucocerebrosidase: α -hexosaminidase ratio (*p* = 0.0014) were significantly lower in cases with dementia than in controls. The pattern of association between *GBA* mutation status and the GCase activity was comparable to the combined samples, suggesting that those with dementia and a neuropathological diagnosis of DLB are driving the association.

### *GBA* mutation carriers show significant differences in lipid species and accumulation of ceramide and sphingolipids

To determine the functional effect of reduced GCase activity in brains with LBs carrying *GBA* mutations compared to those without *GBA* mutations, AD, and control brains we performed a lipidomic analysis in postmortem brain tissue (n = 67) obtained from the primary motor cortex (BA4). The *a priori* hypothesis was that LBD *GBA* mutation carriers should show significant differences in ‘specific’ lipid species (substrate and product of GBA hydrolysis) and accumulation of ceramides and sphingolipids compared to those without *GBA* mutations, AD, and control brains. Characteristics of autopsy subjects with lipidomic analysis is provided as supporting data (S3 Table). The cold and frozen PMIs for autopsy tissue used in our analysis is shown in S4 Table.

**Fig 2:**
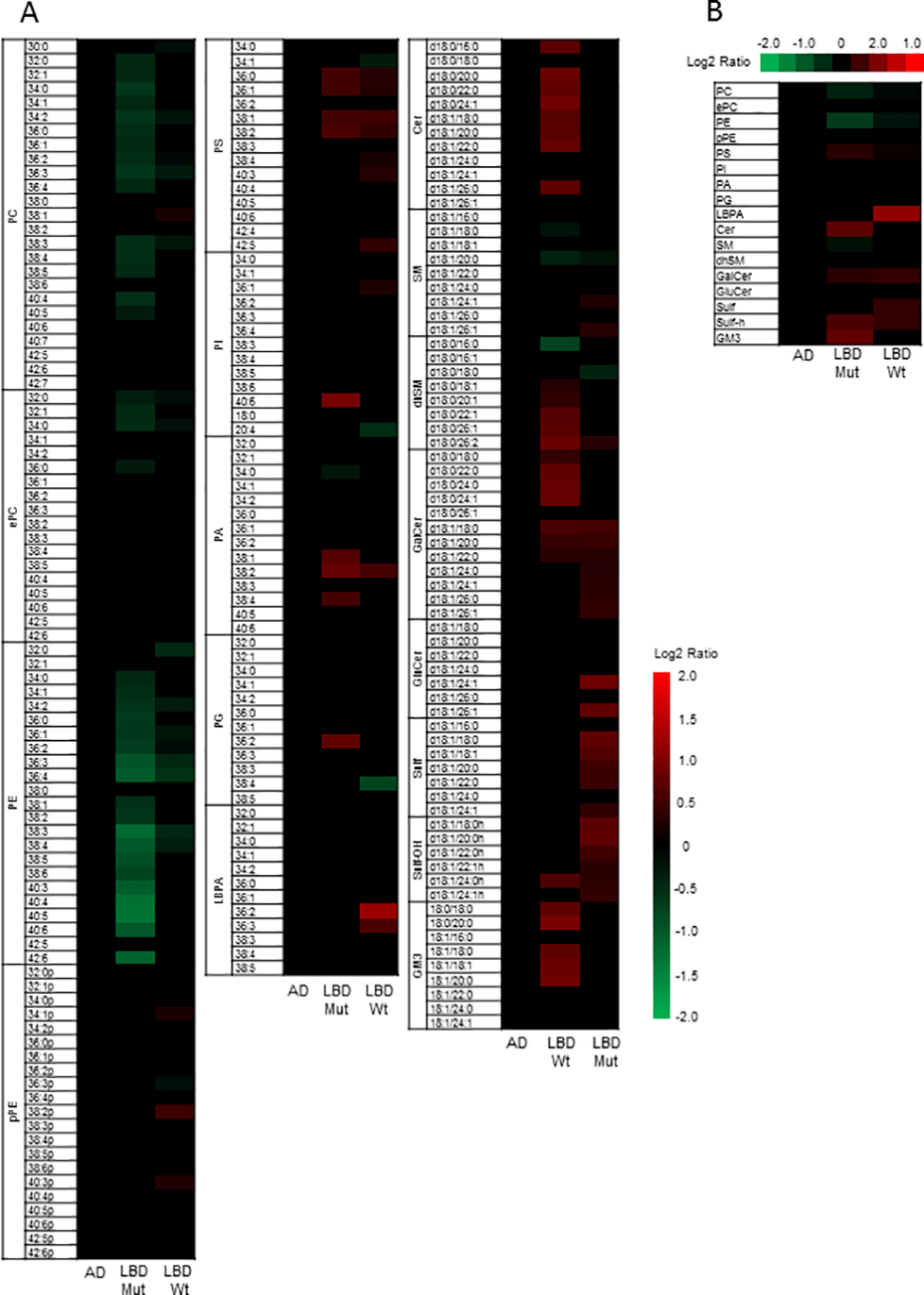
Heat Maps showing significant changes in lipid classes. A) Heat map showing statistically significant changes in major lipid subclasses in LBD *GBA* mutation carriers compared to LBD wildtype, AD cases and controls and **B)** Heat map showing statistically significant changes in lipid classes in LBD *GBA* mutation carriers compared to LBD wildtype, AD cases and controls. The heat map columns reflect all significant lipid changes (*q*<0.05) in a diseased compared to control patients. The color bar represents the log2 value of the ratio of each lipid species. Statistical analysis for the AD and LBD Mutation samples was based on the one way analysis of variance followed by post hoc Fisher’s least significant difference test while the LBD (wildtype) samples was based on Student’s T-test. A false discovery rate control was used to correct for multiple comparisons.

**Fig 3:**
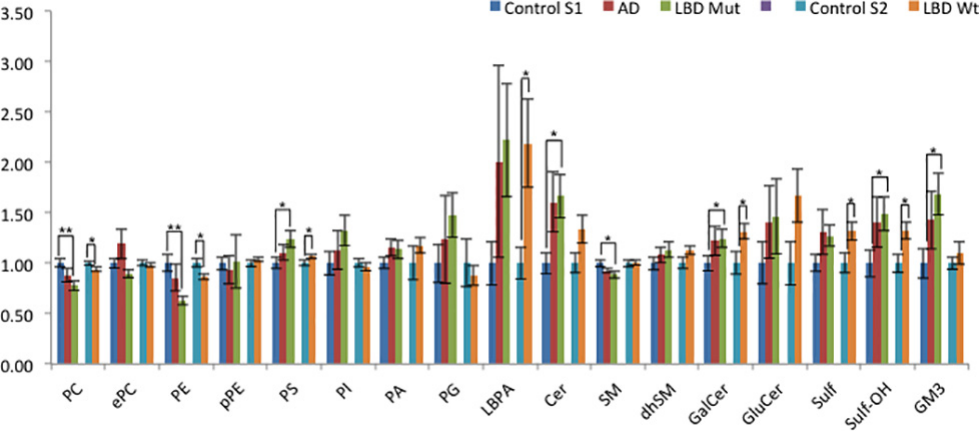
Comparative lipid profile of post-mortem brain tissue obtained from patients diagnosed with various neurological conditions. Comparative lipid profile of post-mortem brain tissue obtained from patients diagnosed with various neurological conditions. The individual lipid subclasses of each group of patients was expressed as relative to control group levels for 2 separate sets of experiments (*i*.*e*. AD and LBD GBA mutation carrier relative to Control S1, LBD non carrier (wildtype) relative to Control S2). Statistical analysis for the AD and LBD Mutation samples was based on the one way analysis of variance followed by post hoc Fisher’s least significant difference test while the LBD non carrier (wildtype) samples was based on Student’s T-test. A false discovery rate control was used to correct for multiple comparisons. * *q*<0.05, ** *q*<0.01, *** *q*<0.001. PC, phosphatidylcholine; ePC, ether phosphatidylcholine; PE, phosphatidylethanolamine; pPE, plasmalogen phosphatidylethanolamine; PS, phosphatidylserine; PI, phosphatidylinositol; PA, phosphatidic acid; PG, phosphatidylglycerol; LBPA, lysobisphosphatidic acid; Cer, ceramide; SM, sphingomyelin; dhSM, dihydrosphingomyelin; GalCer, galactosylceramide; GluCer, glucosylceramide; Sulf, sulfatide; Sulf-h, hydroxylated sulfatide; GM3, monosialodihexosylganglioside

Several lipid classes were significantly altered in brains with LBs carrying *GBA* mutations compared to controls (*P* range: *p*<0.05-*p*<0.01) (Fig 2 and Fig 3) and this remained significant after using an false discovery rate (FDR) control to correct for multiple comparisons of lipids (*q*<0.05-*q*<0.01). Major phospholipid subclasses such as phosphatidylcholine (PC) and phosphatidylethanolamine (PE) were decreased while phosphatidylserine (PS) was increased. There were also striking changes in sphingolipid composition. A small but significant decline in the most abundant sphingolipid sphingomyelin (SM) was seen in the diseased tissue but was compensated by increased levels of select dihydrosphingomyelin (dhSM) species and total ceramide (Cer) levels. While there was a trend towards an increase in accumulation of the known GCase substrate, GluCer, the difference was not statistically significant (Fig 2 and Fig 3). However, the complex glycosphingolipid that is biosynthetically upstream of GluCer, GM3, is highly enriched in these tissues. In addition, there is also a significant accumulation of galactosylceramide (GalCer) and its biosynthetic derivative sulfatides containing hydroxy fatty acid (Sulf-OH).

In brains with LBs without *GBA* mutations, similar changes were observed in major phospholipids PC, PE and PS levels but to a lesser degree compared to those with *GBA* mutations, and in the sphingolipids GalCer, Sulf and Sulf-OH. Interestingly LBD brains with and without *GBA* mutations displayed an accumulation in lysobisphosphatidic acid LBPA (also known as bis(monoacylglycero)phosphate), a lipid that is specifically enriched in the late endosome and lysosome was observed. LBPA also showed a trend for increase in LBD brains with *GBA* mutations. To determine the specificity of our assay and also provide a reference point for lipid changes in the LBD tissue, we also analyzed AD brains and found no significant changes. This is in contrast to a previous study [17], although different brain regions were analyzed (*i*.*e*., prefrontal cortex and entorhinal cortex).

## Discussion

In the current study we performed a genetic analysis of four lysosomal storage disorder genes including *GBA*, *HEXA*, *SMPD1*, *MCOLN1* in 231 brain autopsies from the New York Brain Bank at Columbia University. A biochemical analysis of *GBA* was also performed in a subset of brains. We show that in addition to prior reported variants in *GBA*, variants in *SMPD1* and *MCOLN1* are also significantly associated with LB or ADLBV pathology. Additional gene-wise analyses for variants based on the SKAT algorithm also identified independent association of variants in *GBA*, *SMPD1* and *MCOLN1* that were significantly associated with LBD and ADLBV pathologies but not AD. Strong association and an additive effect of multiple variants in *GBA*+*SMPD1* or *GBA*+*SMPD1*+*MCOLN1* were also observed across all disease phenotypes analysed.

The importance of the lysosomal pathway in CNS function and LBD and PD is highlighted by the identification of genetic risk factors or rare variants/mutations in lysosomal genes in case-control association studies (*GBA* and *NAGLU*)[7,9–11], GWAS studies (*LAMP3*, *SCARB2*)[24,25] or linkage analysis and exome sequencing in PD families (*ATP13A2*, *VPS35* (endolysosomal pathway)[26,27]. A large multisite study of brain autopsy samples from subjects with different forms of dementia identified *GBA* mutations in 7.6% (6/79) of pure DLB cases (OR, 7.6 [95% CI, 1.8–31.9]) compared to 3.6% (8/222) of ADLBV cases (OR, 4.6 [95% CI, 1.2–17.6]) [28].

In our study, multiple variants predicted to be deleterious or damaging in *GBA*, *SMPD1* and *MCOLN1* in autopsy samples were significantly associated with LB and ADLBV pathology. We identified a total of 26 variants in autopsy samples that have been previously reported as mutations in lysosomal storage disorders. These causal mutations in lysosomal storage disorders are usually observed in the homozygous or compound heterozygous state whereas in the autopsy samples that we examined these mutations were observed in the heterozygous state suggesting that haploinsufficiency of lysosomal genes may contribute to LB and ADLBV phenotype. Overall, ~15% of all LBD autopsy samples also carried a variant in more than one lysosomal storage disease gene examined suggesting that ‘multiple hits’ in the same biochemical pathway, the lysosomal pathway, might increase risk for LBD.

Our data also shows that *GBA* mutation status is associated with significantly reduced GCase activity and a neuropathological diagnosis of LBD suggesting that haploinsufficiency or partial enzyme activity leads to increase in α -synuclein levels and Lewy body pathology. A decrease in GCase activity has been reported previously in brain autopsies from patients with Type I Gaucher Disease and parkinsonism and more recently in brain autopsies from patients with PD that carry *GBA* mutations [4,29]. A decrease in GCase activity has also been reported in brain autopsies from patients with sporadic PD without *GBA* mutations, with the greatest reduction in the substantia nigra [29] and conflicting reports of decreased GCase activity in the frontal cortical regions [29, 30]. In LBD brain autopsy samples from the frontal cortex without *GBA* mutations we did not observe a decrease in GCase activity and our findings are consistent with one published study [29]. These conflicting reports of a reduction in GCase activity in different brain regions from PD or LBD autopsy samples without *GBA* mutations may reflect different stages of disease progression, neuronal loss or α -synuclein accumulation.

We have previously demonstrated the utility of lipidomics as a means to understand dysregulation of lipid metabolism and generate novel insights linked to AD pathogenesis[17]. Applying similar methodologies to the analysis of the motor cortex region of LBD brains with and without *GBA* mutations, we observed that there are significant alterations in both major phospholipid and sphingolipid subclasses compared to controls. In the case of LBD carrying *GBA* mutations, GluCer was not significantly accumulated as one might expect. This may be reconciled by the existence of non-lysosomal glucosylceramidase *GBA2* that is significantly expressed in the brain and can compensate for the deficiency in *GBA* activity [31, 32]. Nevertheless, it is worth noting that in both cases of LBD with and without *GBA* mutation, there appears to be significant or a trend towards accumulation of other sphingolipid subclasses including Cer, GalCer, Sulf, Sulf-OH and GM3 and the unusual phospholipid LBPA. LBPA is enriched in late endosomes where it functions in biogenesis of multivesicular bodies [33] and also in lysosomes where it plays a role in stimulating the hydrolysis of membrane bound sphingolipids. The overall profile of both LBD with and without *GBA* mutation cases suggests a common theme of dysfunction occurring in the endolysosomal degradative pathway that ultimately lead to defects in lysosomal clearance of autophagosomes and an accumulation of α -synuclein in LBD.

Recent studies in AD suggest that disease pathogenesis may begin more than 20 years before the onset of dementia [34]. Similarly in LBD, non-motor symptoms may predate motor symptoms by decades suggesting pathophysiological changes before clinical onset [34–37]. To date, there are no effective biomarkers for LBD. Our study suggests that combined genetic and lipidomic data may prove effective in disease risk prediction, biomarker development (CSF) and targeted therapeutic strategies.

## Supporting Information

S1 Methods (DOCX)

S1 Table Demographic and Neuropathological Characteristics of White Autopsy Subjects. *APOE4 missing for 16 cases
(DOCX)

S2 Table Gene-wise association SKAT analysis with AJ controls. A) Gene-wise association with SKAT analysis with AJ controls only (n = 128) and B) Gene wise association SKAT analysis with brain controls (n = 33) and AJ controls (n = 128). *Corrected for covariates. ** Indicates number of markers included in the test. ^**1**^ Risk variants are variants more frequent among cases than controls; whereas, variants are considered protective when they are more frequent in controls than cases.
(DOCX)

S3 Table Characteristics of Autopsy Subjects who had Lipidomic Analysis. * APOE missing in 2 cases.
(DOCX)

S4 Table Cold and Frozen PMIs brain autopsies. (DOCX)
