# Supplementary material for: Gene-Wise Association of Variants in Four Lysosomal Storage Disorder Genes in Neuropathologically Confirmed Lewy Body Disease
Source: PLoS One. 2015 May 1;10(5):e0125204. doi: 10.1371/journal.pone.0125204 (PMC4416714; doi:10.1371/journal.pone.0125204)
Supplement: S1 Methods — (DOCX) [file pone.0125204.s001.docx]

**Supplementary Methods**

**Brain Material**

*Neuropathological evaluation*

LB pathology was assessed according to the Third Report of the DLB consortium, and utilized α-synuclein immunohistochemistry, with LB presence characterized as brainstem-predominant, or “cortical” (limbic or neocortical)[[1](#_ENREF_1)]. Alzheimer’s plaque and tangle pathology was detected using H&E and Bielschowsky stains, and β-amyloid and AT-8 immunohistochemistry, and rated using Braak & Braak, CERAD, and NIA-Reagan Institute (NIA-RI) criteria[[2](#_ENREF_2)]. All cases with Braak stage III, IV, V, or VI neurofibrillary pathology, and/or plaque-based CERAD possible, probable, or definite AD, were rated as having “any Alzheimer’s pathology”. Neuropathological findings were described as per the National Alzheimer’s Coordinating Center (NACC) Neuropathology Manual V8.0[[3](#_ENREF_3)]. Only cases that met NIA-RI criteria for intermediate or high likelihood of AD were deemed to have an “AD pathological diagnosis”. Cases with cortical LB by consortium criteria, were termed DLB if they did not have concomitant AD pathological diagnosis, and were termed Lewy Body Variant of AD (ADLBV) if they did.

*Clinical evaluation of dementia*

Dementia was determined by consensus conference using DSM-IV-TR criteria; AD was determined using NINDS-ADRDA criteria, and LBD using McKeith criteria. For cases not seen in proximity to death, clinical history was re-obtained. As such, “onset of dementia” age was generally obtained prospectively during evaluations, but occasionally retrospectively in cases for whom there was more than a year between last clinical evaluation and death/autopsy.

**Population Stratification and Ashkenazi Jewish Ancestry**

Since a founder effect for LSD gene mutations have been reported in the AJ population we also determined AJ ancestry in brain autopsy samples. Information about AJ ancestry was not available for brain autopsies. We used two methods to examine AJ ancestry and underlying population structure in brain autopsies. In the first method, Multidimensional scaling (MDS) as implemented in the program PLINK (Version 1.07) for detecting population outliers and adjusting for population stratification was used. Briefly, we used 288, 963 autosomal SNPs for brain autopsies (n=62), augmented with 252 AJ samples with subjects from the HapMap website (http:www.hapmap.org/), which included 90 CEU, 90 Yorubans and 90 Asians. The best fitting model assumed two underlying populations with overlap of 17 white brain autopsies with the AJ cluster and the remainder of the white brain autopsies with the white CEU cluster. In the second method, principle component analysis (PCA) as implemented in the GCTA package was used to examine ancestry and admixture in white brain autopsies, AJ samples together with subjects from HapMap. Projection of all the sample genotypes along the two principle components (PC2 and PC3) is shown in Supplementary Figure 1. As in the MDS analysis performed in PLINK, there is tight clustering of 27 brain autopsies with AJ sample cluster and the remainder of the white brain autopsies cluster with CEU samples.

**Supplementary Figure 1 PCA to examine AJ ancestry**

**A) All autopsies with GWAS data (n=62)**

**B) White autopsies with GWAS data (n=49)**

**AJ Control Samples**

A total of 128 Ashkenazi Jewish (AJ) healthy controls were used in a secondary analysis to supplement autopsy controls.

We now include data for 128 AJ population healthy controls. High depth whole genome sequencing was performed in 128 healthy controls and an AJ reference panel developed. Compared to a European reference panel, our AJ panel is 47% richer in novel variants and 8-fold more effective at filtering benign variants, which is necessary for interpreting AJ clinical genomes. The demographic and medical characteristics of the 128 sequenced individuals is summarized below. Further details of the cohort including sample selection is described in Carmi et al (2014)[[4](#_ENREF_4)] .

| **Trait (Atzmon’s lab)** | **Mean ± STDEV** | **Trait (Clark’s lab)** | **Mean ± STDEV** |
| --- | --- | --- | --- |
| **All (n)** | 74 | **All (n)** | 54 |
| **Female (n)** | 45 | **Female (n)** | 33 |
| **Male (n)** | 29 | **Male (n)** | 21 |
| **Age (years)** | 68.8±7.7 (range 49-85) | **Age (years)** | 68.7±10.4 (range 39-88) |
| **Cholesterol (mg/dL)** | 200±42.1 | **Intellectual impairment (n)** | 1 |
| **Triglycerides (mg/dL)** | 132±71.4 | **Thought disorder (n)** | 1 |
| **HDL (mg/dL)** | 65.4±17.2 | **Depression (n)** | 2 |
| **LDL (mg/dL)** | 108±34.4 | **Family history of PD in first degree relatives (conservative) (n)** | 2 |
| **Glucose (mg/dL)** | 81.9±14.6 | **Family history of AD in first degree relatives (conservative) (n)** | 3 |
| **Waist circumference (inch)** | 35.1±6.9 | **Total mMMS Score** | 56.2±1.4 |
| **Body Mass Index (kg/m^2^)** | 26.4±5.1 | **Total UPDRS part II score** | 0.25±0.85 |
| **Systolic Blood pressure (mm Hg)** | 139±20.6 | **Total UPDRS part III score** | 1.81±3.20 |
| **Diastolic Blood pressure (mm Hg)** | 79.6±11.2 |  | |

*Demographic and medical characteristics of the AJ samples.* For description of the cohorts (Atzmon’s lab and Clark’s lab), see Carmi et al (2014). Except the gender and the mMMS score, all traits in the Clark’s lab cohort were computed over 53 samples. PD: Parkinson’s disease. AD: Alzheimer’s disease. The mMMS score is calculated from a modification of the modified Mini-Mental State Examination, with a maximum score of 57 (computed over 15 samples). The Unified Parkinson’s Disease Rating Scale (UPDRS) parts II and III contain 44 questions each measured on a 5-point scale (0-4).

**Molecular Genetic Analysis**

*Analysis of functional effect of variants*

Condel uses a consensus deleteriousness score that combines various tools (SIFT, Polyphen2, MAPP LogR Pfam E-value and Mutation assessor). The scores of different methods are weighted using the complementary cumulative distributions produced by the five methods on a dataset of approximately 20000 missense SNPs, both deleterious and neutral. The probability that a predicted deleterious mutation is not a false positive of the method and the probability that a predicted neutral mutation is not a false negative are employed as weights[[5](#_ENREF_5)].

**Enzyme Activity Measurements**

Sample triplicates each containing 50 u g of protein (brain homogenates) were used to measure enzyme activity in autopsy tissue (Cerebellum, BA4 and BA9 and ScxV). Enzyme activity was determined using either 4-methylumbelliferyl-β-D-glucopyranoside (4-MUG)( β-glucocerebrosidase) or 4-methylumbelliferyl-2-acetoamido-2-deoxy-b-D-glucopyranoside (4MUGlcNAc) (Hexosaminidase A) as substrate as previously described[[6](#_ENREF_6),[7](#_ENREF_7)]. Brain autopsy tissue (Cerebellum, BA4 and BA9 and ScxV) samples were homogenized in water (10% wt./vol.) using a Misonix Sonic Dismembrator and centrifuge at 30,000 Xg for 20 min.  Protein concentration was determined using the Lowry method. The reaction mixture for β-glucocerebrosidase determination consisted of 50ug of protein, 50ul of 20mM 4-methylumbelliferyl-β-D-glucopyranoside, 10ul of 1M Citrate-Phosphate pH 5.0 and 10ul of 2% Sodium Tauro Deoxycholate. The reaction mixture was incubated at 37°C for 2Hours and then subsequently stopped with 2 ml 0.2 M glycine buffer, pH 10.3.  The Hexosamindase A enzymatic reaction mixture consisted of 10ug of protein and 100ul of 3mM 4-methylumbelliferyl-2-acetoamido-2-deoxy-b-D-glucopyranoside in Citrate-Phosphate buffer pH4.0. Samples were incubated at 37°C for 10min and 0.2M glycine buffer was also used to stop the reaction. Fluorescence was determined in fluorescence spectrophotometer (Hitachi F-2500) at an excitation wavelength of 365 nm and emission wavelength of 448 nm. Samples were compared against a 4-methylumbelliferone (4-MU) standard curve prepared in 0.2 M glycine buffer. Enzyme activities were calculated in nmoles of 4-MU hydrolyzed/mg protein/hr. LBD brains did not carry variants in any of the other LSD genes analyzed. Frozen post-mortem interval (PMI) was available for all autopsy tissue and PMI did not appear to influence GCase activity.

**Lipid Profiling**

Lipid extracts were prepared using a modified Bligh/Dyer extraction procedure, spiked with appropriate internal standards including PC (14:0/14:0), PE (14:0/14:0), PS (14:0/14:0), PA (14:0/14:0), PG (15:0/15:0), LBPA (14:0/14:0), Cer (d18:1/17:0), GalCer (d18:1/12:0), GluCer (d18:1/12:0), Sulf (d18:1/12:0) and SM (d18:1/12:0) obtained from Avanti Polar Lipids (Alabaster, AL) and PI (16:0/16:0) obtained from Echelon Biosciences (Salt Lake City, UT)[[8](#_ENREF_8)]. The samples were analyzed using an Agilent 1260 HPLC system coupled to an Agilent 6490 Triple Quadrupole mass spectrometer. Separation of individual phospholipid and sphingolipid subclasses by normal phase HPLC was carried out using a Phenomenex Luna Si column (i.d. 2.0x100 mm, 3µm) or Agilent Rx-Sil column (i.d. 2.1x100mm, 1.8µm) with the following conditions: mobile phases A (chloroform: methanol: ammonium hydroxide, 89.8:10:0.2) and B (chloroform: methanol: ammonium hydroxide: water, 55:39:0.2:5.8); flow rate of 0.3 ml/min; 5% B for 2 min, then linearly changed to 70% B over 18 min and maintained for 3 min. The column was re-equilibrated for the next sample by changing the gradient back to 5% B over 2 min and maintained for 6 min for column re-equilibration. Multiple reaction monitoring transitions were set up for quantitative analysis of various lipid species and referenced to the spiked internal standard concentrations as done previously[[8](#_ENREF_8),[9](#_ENREF_9)]. The lipid levels for each sample was calculated by summing up the total number of moles of all lipid species measured, and then normalizing that total to give mol %. The final data are presented as mean mol % with error bars showing standard error of means.

**Reference**

1. McKeith IG, Dickson DW, Lowe J, Emre M, O'Brien JT, Feldman H, et al. Diagnosis and management of dementia with Lewy bodies: third report of the DLB Consortium. Neurology. 2005;65: 1863-1872.

2. Mirra SS, Heyman A, McKeel D, Sumi SM, Crain BJ, Brownlee LM, et al. The Consortium to Establish a Registry for Alzheimer's Disease (CERAD). Part II. Standardization of the neuropathologic assessment of Alzheimer's disease. Neurology. 1991;41: 479-486.

3. National Alzheimer’s Coordinating Center. NACC Neuropathology (NP) Diagnosis Coding Guidebook. Seattle: University of Washington: 2005-2008.

4. Carmi S, Hui KY, Kochav E, Liu X, Xue J, Grady F, et al. Sequencing an Ashkenazi reference panel supports population-targeted personal genomics and illuminates Jewish and European origins. Nat Commun. 2014;5: 4835-4835.

5. González-Pérez A, López-Bigas N. Improving the Assessment of the Outcome of Nonsynonymous SNVs with a Consensus Deleteriousness Score, Condel. Am J Hum Genet. 2011;88: 440-449.

6. Takagi Y, Kriehuber E, Imokawa G, Elias PM, Holleran WM. Beta-glucocerebrosidase activity in mammalian stratum corneum. J Lipid Res. 1999;40: 861-869.

7. Balducci C, Pierguidi L, Persichetti E, Parnetti L, Sbaragli M, Tassi C, et al. Lysosomal hydrolases in cerebrospinal fluid from subjects with Parkinson's disease. Mov Disord. 2007; 22: 1481-1484.

8. Chan RB, Oliveira TG, Cortes EP, Honig LS, Duff KE, Small SA, et al. Comparative lipidomic analysis of mouse and human brain with Alzheimer disease. J Biol Chem. 2012;287: 2678-2688.

9. Chan R, Uchil PD, Jin J, Shui G, Ott DE, Mothes W, et al. Retroviruses human immunodeficiency virus and murine leukemia virus are enriched in phosphoinositides. J Virol. 2008;82: 11228-11238.
