# Supplementary material for: Gene-Wise Association of Variants in Four Lysosomal Storage Disorder Genes in Neuropathologically Confirmed Lewy Body Disease
Source: PLoS One. 2015 May 1;10(5):e0125204. doi: 10.1371/journal.pone.0125204 (PMC4416714; doi:10.1371/journal.pone.0125204)
Supplement: S1 Table — *APOE4 missing for 16 cases (DOCX) [file pone.0125204.s002.docx]

**S1 Table Demographic Characteristics of White Autopsy Subjects**

| **All autopsies** | | **LBD** | **ADLBV** | **AD** | **Control** | **Total** |
| --- | --- | --- | --- | --- | --- | --- |
|  | **N** | 56 | 59 | 61 | 20 | 196 |
| **Male** | **%** | 73.2 | 57.6 | 39.3 | 50.0 | 55.6 |
| **Age at Dementia (yr)** | **Mean** | 67.2 | 68.3 | 70.8 |  | 68.8 |
|  | **SD** | 10.2 | 9.8 | 8.2 |  | 9.5 |
| **Age at Death (yr)** | **Mean** | 78.2 | 78.4 | 81.4 | 70.8 | 78.5 |
|  | **SD** | 8.6 | 8.4 | 8.6 | 15.0 | 9.8 |
| **Duration (yr)** | **Mean** | 11.2 | 9.2 | 10.0 |  | 10.1 |
|  | **SD** | 5.8 | 4.6 | 4.1 |  | 4.9 |
| **Education (yr)** | **Mean** | 16.6 | 14.6 | 14.8 | 16.2 | 15.3 |
|  | **SD** | 2.4 | 4.1 | 3.5 | 1.0 | 3.5 |
| **LB Pathology Present** | **%** | 100.0 | 100.0 | 19.7 | 5.0 | 65.3 |
| **LB Cortical Pathology Present** | **%** | 100.0 | 100.0 | 0.0 | 0.0 | 58.7 |
| **LB Subcortical Pathology Present** | **%** | 66.1 | 66.1 | 14.8 | 0.0 | 43.4 |
| **AD Pathology Present** | **%** | 78.6 | 100.0 | 100.0 | 35.0 | 87.2 |
| **AD Pathological Diagnosis** | **%** | 0.0 | 100.0 | 100.0 | 0.0 | 61.2 |
| **GBA mutation** | **N(Individuals)** | 27 | 14 | 5 | 1 | 47 |
|  | **%** | 48.2 | 23.7 | 8.2 | 5.0 | 24.0 |
| **SMPD1 mutation** | **N(Individuals)** | 11 | 12 | 4 | 2 | 29 |
|  | **%** | 19.6 | 20.3 | 6.6 | 10.0 | 14.8 |
| **HEXA mutation** | **N(Individuals)** | 8 | 15 | 8 | 6 | 37 |
|  | **%** | 14.3 | 25.4 | 13.1 | 30.0 | 18.9 |
| **MCOLN1 mutation** | **N(Individuals)** | 17 | 17 | 16 | 5 | 55 |
|  | **%** | 30.4 | 28.8 | 26.2 | 25.0 | 28.1 |
| **APOE (no E4)*** | **N(Individuals)** | 31 | 25 | 20 | 16 | 92 |
|  | **%** | 55.4 | 42.4 | 32.8 | 80.0 | 39.8 |
| **APOE (one E4)*** | **N(Individuals)** | 12 | 20 | 20 | 1 | 53 |
|  | **%** | 21.4 | 33.9 | 32.8 | 5.0 | 27.0 |
| **APOE (two E4)*** | **N(Individuals)** | 4 | 10 | 10 | 1 | 25 |
|  | **%** | 7.1 | 16.9 | 16.4 | 5.0 | 12.8 |

*APOE4 missing for 16 cases
