# Supplementary material for: Gene-Wise Association of Variants in Four Lysosomal Storage Disorder Genes in Neuropathologically Confirmed Lewy Body Disease
Source: PLoS One. 2015 May 1;10(5):e0125204. doi: 10.1371/journal.pone.0125204 (PMC4416714; doi:10.1371/journal.pone.0125204)
Supplement: S2 Table — A) Gene-wise association with SKAT analysis with AJ controls only (n = 128) and B) Gene wise association SKAT analysis with brain controls (n = 33) and AJ controls (n = 128). *Corrected for covariates. ** Indicates number of markers included in the test. 1 Risk variants are variants more frequent among cases than controls; whereas, variants are considered protective when they are more frequent in controls than cases. (DOCX) [file pone.0125204.s003.docx]

**S2 Table Gene wise association SKAT analysis with AJ controls**

**a) Gene wise association SKAT analysis with AJ controls (n=128)***

| **Sample size Gene** | **LBD vs. AJ controls (n=59 vs.128)** | | **ADLBV vs. AJ controls (n=68 vs.128)** | | **AD vs. AJ controls (n=71 vs.128)** | |
| --- | --- | --- | --- | --- | --- | --- |
|  | **P value** | **Marker (n)^**^** | **P value** | **Marker (n)^**^** | **P value** | **Marker (n)^**^** |
| **All variants** | | | | | | |
| **GBA** | 1.57x10^-9^ | 9 | 5.92x10^-3^ | 6 | 8.73x10^-3^ | 4 |
| **SMPD1** | 6.87x10^-5^ | 11 | 3.08x10^-5^ | 10 | 2.56x10^-6^ | 9 |
| **HEXA** | 0.282 | 8 | 4.50x10^-2^ | 10 | 0.174 | 6 |
| **MCOLN1** | 0.166 | 7 | 4.20x10^-2^ | 11 | 7.38x10^-2^ | 7 |
| **GBA+SMPD1** | 1.75x10^-12^ | 20 | 7.14x10^-6^ | 16 | 9.77x10^-7^ | 13 |
| **GBA+SMPD1+MCOLN1** | 1.31x10^-9^ | 27 | 7.43x10^-6^ | 27 | 4.08x10^-7^ | 20 |
| **Risk variants^1^** | | | | | | |
| **GBA** | 1.22x10^-9^ | 8 | 6.23x10^-3^ | 5 | 3.02x10^-3^ | 2 |
| **SMPD1** | 1.97x10^-5^ | 7 | 7.70x10^-7^ | 5 | 1.75x10^-5^ | 4 |
| **HEXA** | 4.26x10^-2^ | 5 | 1.78x10^-4^ | 8 | 0.573 | 3 |
| **MCOLN1** | 0.114 | 1 | 1.95x10^-4^ | 5 | 7.05x10^-2^ | 2 |
| **GBA+SMPD1** | 2.12x10^-13^ | 15 | 1.31 x10^-8^ | 10 | 7.87x10^-7^ | 6 |
| **GBA+SMPD1+MCOLN1** | 4.27x10^-13^ | 16 | 2.04 x10^-11^ | 15 | 2.64x10^-6^ | 8 |
| **Protective variants^1^** | | | | | | |
| **GBA** | 1.96x10^-6^ | 1 | 1.91x10^-6^ | 1 | 0.664 | 2 |
| **SMPD1** | 1.32x10^-2^ | 4 | 0.102 | 5 | 2.25x10^-3^ | 5 |
| **HEXA** | 0.130 | 3 | 0.350 | 2 | 7.72x10^-2^ | 3 |
| **MCOLN1** | 8.28x10^-2^ | 6 | 1.85x10^-2^ | 6 | 2.78x10^-3^ | 5 |
| **GBA+SMPD1** | 1.20x10^-2^ | 5 | 9.3 x10^-2^ | 6 | 1.46x10^-3^ | 7 |
| **GBA+SMPD1+MCOLN1** | 5.67x10^-3^ | 11 | 3.90x10^-3^ | 12 | 2.98x10^-5^ | 12 |

*Corrected for covariates. ** Indicates number of markers included in the test.

**^1^** Risk variants are variants more frequent among cases than controls; whereas, variants are considered protective when they are more frequent in controls than cases.

**b) Gene wise association SKAT analysis with brain controls (n=33) and AJ controls (n=128)***

| **Sample size Gene** | **LBD vs. brain controls +AJ controls**  **(n=59 vs 161)** | | **ADLBV vs. brain controls +AJ controls (n=68 vs 161)** | | **AD vs. brain controls +AJ controls (n=71 vs 161)** | |
| --- | --- | --- | --- | --- | --- | --- |
|  | **P value** | **Marker (n)^**^** | **P value** | **Marker (n)^**^** | **P value** | **Marker (n)^**^** |
| **All variants** | | | | | | |
| **GBA** | 1.27x10^-9^ | 11 | 7.81x10^-3^ | 7 | 6.76x10^-2^ | 5 |
| **SMPD1** | 1.07x10^-5^ | 13 | 1.37x10^-5^ | 12 | 4.37x10^-6^ | 11 |
| **HEXA** | 0.189 | 9 | 5.69x10^-2^ | 11 | 0.172 | 7 |
| **MCOLN1** | 0.231 | 8 | 7.87x10^-2^ | 11 | 0.155 | 8 |
| **GBA+SMPD1** | 1.48x10^-12^ | 24 | 1.03x10^-5^ | 19 | 2.58x10^-6^ | 16 |
| **GBA+SMPD1+MCOLN1** | 5.66x10^-10^ | 32 | 6.13x10^-6^ | 30 | 1.16x10^-6^ | 24 |
| **Risk variants^1^** | | | | | | |
| **GBA** | 6.02x10^-11^ | 10 | 7.02x10^-3^ | 6 | 1.56x10^-2^ | 3 |
| **SMPD1** | 2.84x10^-6^ | 7 | 4.8 x10^-7^ | 5 | 4.59x10^-6^ | 4 |
| **HEXA** | 4.28x10^-3^ | 4 | 8.70x10^-5^ | 8 | 0.257 | 2 |
| **MCOLN1** | 6.79x10^-2^ | 1 | 1.03x10^-2^ | 6 | 5.02x10^-2^ | 2 |
| **GBA+SMPD1** | 7.87x10^-16^ | 17 | 1.08x10^-9^ | 11 | 6.64x10^-7^ | 7 |
| **GBA+SMPD1+MCOLN1** | 7.67 x10^-15^ | 18 | 3.21x10^-10^ | 17 | 1.86x10^-6^ | 9 |
| **Protective variants^1^** | | | | | | |
| **GBA** | 1.39x10^-4^ | 1 | 7.81x10^-5^ | 1 | 0.564 | 2 |
| **SMPD1** | 1.97x10^-2^ | 6 | 0.104 | 7 | 2.82x10^-3^ | 7 |
| **HEXA** | 0.157 | 5 | 0.396 | 3 | 6.77x10^-2^ | 5 |
| **MCOLN1** | 0.115 | 7 | 3.53x10^-3^ | 5 | 9.22x10^-3^ | 6 |
| **GBA+SMPD1** | 1.67x10^-2^ | 7 | 8.97x10^-2^ | 8 | 1.99x10^-3^ | 9 |
| **GBA+SMPD1+MCOLN1** | 6.62x10^-3^ | 14 | 1.97x10^-3^ | 13 | 1.99x10^-3^ | 9 |

*Corrected for covariates. ** Indicates number of markers included in the test.

**^1^** Risk variants are variants more frequent among cases than controls; whereas, variants are considered protective when they are more frequent in controls than cases.
