# Supplementary material for: Gene-Wise Association of Variants in Four Lysosomal Storage Disorder Genes in Neuropathologically Confirmed Lewy Body Disease
Source: PLoS One. 2015 May 1;10(5):e0125204. doi: 10.1371/journal.pone.0125204 (PMC4416714; doi:10.1371/journal.pone.0125204)
Supplement: S3 Table — * APOE missing in 2 cases. (DOCX) [file pone.0125204.s004.docx]

**S3 Table Characteristics of Autopsy Subjects who had Lipidomic Analysis**

| **Autopsies with Lipidomics** | | **LBD** | **ADLBV** | **AD** | **PD** | **Control** | **Total** |
| --- | --- | --- | --- | --- | --- | --- | --- |
|  | **N** | **34** | **3** | **4** | **9** | **17** | **67** |
| **Male** | **%** | 73.5% | 66.7% | 75.0% | 33.3% | 47.1% | 61.2% |
| **Age at Dementia (yr)** | **Mean** | 66.1 | 68.5 | 74.7 | 64.5 |  | 67.0 |
|  | **SD** | 10.3 | 3.5 | 10.1 | 21.9 |  | 10.6 |
| **Age at Death (yr)** | **Mean** | 77.9 | 79.5 | 85.6 | 81.5 | 73.7 | 77.8 |
|  | **SD** | 8.9 | 4.3 | 4.9 | 7.8 | 13.6 | 10.2 |
| **Duration (yr)** | **Mean** | 11.1 | 9.5 | 12.9 | 15.9 |  | 11.5 |
|  | **SD** | 6.1 | 2.1 | 7.1 | 15.7 |  | 6.5 |
| **Education (yr)** | **Mean** | 16.5 | 15.0 | 17.3 | 17.6 | 12.8 | 16.1 |
|  | **SD** | 2.3 | 5.6 | 3.6 | 2.2 | 4.3 | 3.2 |
| **Ethnicity** | **% White** | 94.1 | 100 | 100 | 77.8 | 52.9 | 82.1 |
| **LB Pathology Present** | **%** | 100 | 100 | 75.0 | 100 | 0 | 73.1 |
| **LB Cortical Pathology Present** | **%** | 100 | 100 | 0 | 0 | 0 | 55.2 |
| **LB Subcortical Pathology Present** | **%** | 55.9 | 100 | 50.0 | 66.7 | 0 | 44.8 |
| **AD Pathology Present** | **%** | 82.4 | 100 | 100 | 66.7 | 41.2 | 71.6 |
| **AD Pathological Diagnosis** | **%** | 0 | 100 | 100 | 0 | 0 | 10.4 |
| **GBA mutation** | **N(Individuals)** | 14 | 0 | 0 | 0 | 1 | 15 |
|  | **%** | 41.2 | 0 | 0 | 0 | 6.3 | 22.7 |
| **SMPD1 mutation** | **N(Individuals)** | 4 | 1 | 2 | 1 | 1 | 9 |
|  | **%** | 11.8 | 33.3 | 50.0 | 11.1 | 6.3 | 13.6 |
| **HEXA mutation** | **N(Individuals)** | 3 | 1 | 1 | 3 | 4 | 12 |
|  | **%** | 8.8 | 33.3 | 25.0 | 33.3 | 25.0 | 18.2 |
| **MCOLN1 mutation** | **N(Individuals)** | 4 | 1 | 4 | 3 | 4 | 16 |
|  | **%** | 11.8 | 33.3 | 100 | 33.3 | 25.0 | 24.2 |
| **APOE (no E4)*** | **N(Individuals)** | 21 | 2 | 2 | 9 | 14 | 48 |
|  | **%** | 63.6 | 66.7 | 50.0 | 100 | 87.5 | 73.8 |
| **APOE (one E4)*** | **N(Individuals)** | 9 | 0 | 2 | 0 | 1 | 12 |
|  | **%** | 27.3 | 0.0 | 50.0 | 0 | 6.3 | 18.5 |
| **APOE (two E4)*** | **N(Individuals)** | 3 | 1 | 0 | 0 | 1 | 5 |
|  | **%** | 9.1 | 33.3 | 0 | 0 | 6.3 | 7.7 |

* APOE missing in 2 cases
