# Supplementary material for: Gene-Wise Association of Variants in Four Lysosomal Storage Disorder Genes in Neuropathologically Confirmed Lewy Body Disease
Source: PLoS One. 2015 May 1;10(5):e0125204. doi: 10.1371/journal.pone.0125204 (PMC4416714; doi:10.1371/journal.pone.0125204)
Supplement: S4 Table — (DOCX) [file pone.0125204.s005.docx]

| **Deidentified sample ID** | **Cold PMI** | **Frozen PMI** | **Block** |
| --- | --- | --- | --- |
| 1 | 13:30 | 16:05 | SBB2.0-BA0004 |
| 2 | 5:35 | 6:50 | SBB2.1-BA0004 |
| 3 | 2:24 | 9:14 | SBB2.1-BA0004 |
| 4 | None listed | 18:55 | SBB2.1-BA0004 |
| 5 | 10:25 | 21:55 | SBB2.1-BA0004 |
| 6 | 3:00 | 10:53 | SBB2.1-BA0004 |
| 7 | 13:55 | 15:05 | SBB2.5-BA0004 |
| 8 | 5:50 | 7:00 | SBB2.3-BA0004 |
| 9 | 5:10 | 6:40 | SBB2.1-BA0004 |
| 10 | 3:15 | 6:40 | SBB2.4-BA0004 |
| 11 | 21:02 | 23:42 | SBB2.1-BA0004 |
| 12 | 15:12 | 16:47 | SBB2.3-BA0004 |
| 13 | 4:47 | 7:17 | SBB2.1-BA0004 |
| 14 | 20:50 | 40:45 | SBB2.1-BA0004 |
| 15 | 4:16 | 8:16 | SBB2.7-BA0004 |
| 16 | None listed | 23:45 | SBB2.1-BA0004 |
| 17 | 3:36 | 5:23 | SBB2.1-BA0004 |
| 18 | 2:20 | 4:05 | SBB2.7-BA0004 |
| 19 | 2:00 | 16:55 | SBB2.1-BA0004 |
| 20 | 4:15 | 24:20 | SBB2.1-BA0004 |
| 21 | 3:00 | 15:13 | SBB2.2-BA0004 |
| 22 | 2:40 | 13:20 | SBB2.1-BA0004 |
| 24 | 10:30 | 11:40 | SBB2.2-BA0004 |
| 25 | NaN | NaN | SBB2.3-BA0004 |
| 26 | 3:41 | 5:31 | SBB2.1-BA0004 |
| 27 | 3:30 | 4:40 | SBB2.2-BA0004 |
| 28 | 1:40 | 14:10 | SBB2.1-BA0004 |
| 29 | 1:55 | 6:55 | SBB2.1-BA0004 |
| 30 | 5:15 | 7:40 | SBB2.1-BA0004 |
| 31 | 4:55 | 7:00 | SBB2.1-BA0004 |
| 32 | 4:10 | 28:55 | SBB2.1-BA0004 |
| 33 | 4:55 | 6:05 | SBB2.1-BA0004 |
| 34 | 4:15 | 15:20 | SBB2.1-BA0004 |
| 35 | 3:30 | 12:25 | SBB2.5-BA0004 |
| 36 | 6:02 | 7:22 | SBB2.1-BA0004 |
| 37 | 1:45 | 13:55 | SBB2.2-BA0004 |
| 38 | 4:58 | 32:33 | SBB2.1-BA0004 |
| 39 | 5:30 | 25:50 | SBB2.1-BA0004 |
| 40 | 18:45 | 28:00 | SBB2.1-BA0004 |
| 41 | 5:39 | 20:51 | SBB2.1-BA0004 |
| 42 | 7:40 | 9:25 | SBB2.1-BA0004 |
| 43 | 5:20 | 8:00 | SBB2.1-BA0004 |
| 44 | 4:04 | 18:18 | SBB2.2-BA0004 |
| 45 | 2:10 | 18:50 | SBB2.1-BA0004 |
| 46 | 5:00 | 11:00 | SBB2.3-BA0004 |
| 47 | 7:30 | 9:50 | SBB2.3-BA0004 |
| 48 | 11:55 | 27:15 | SBB2.3-BA0004 |
| 49 | 3:30 | 8:55 | SBB2.8-BA0004 |
| 50 | 6:44 | 13:34 | SBB2.8-BA0004 |
| 51 | 6:28 | 8:23 | SBB2.9-BA0004 |
| 52 | 17:30 | 26:20 | SBB2.09-BA0004 |
| 53 | 9:55 | 11:05 | SBB2.2-BA0004 |
| 54 | 7:15 | 8:53 | SBB2.2-BA0004 |
| 55 | 3:53 | 6:26 | SBB2.9-BA0004 |
| 56 | 11:45 | 13:35 | SWmV2.0-BA0004/SCxV2.9-BA0004 |
| 57 | 4:11 | 61:36 | SBB2.1-BA0004 |
| 58 | 4:40 | 22:05 | SBB2.2-BA0004 |
| 59 | 3:15 | 10:05 | SBB2.2-BA0004 |
| 60 | 20:00 | 22:00 | SBB2.2-BA0004 |
| 61 | 0:30 | 18:20 | SBB2.5-BA0004 |
| 62 | 5:15 | 30:30 | SBB2.5-BA0004 |
| 63 | 9:45 | 12:50 | SBB2.4-BA0004 |
| 64 | 1:10 | 29:40 | SBB2.5-BA0004 |
| 65 | 16:36 | 17:48 | SBB2.4-BA0004 |
| 66 | 9:20 | 29:37 | SBB2.2-BA0004 |
| 67 | 7:16 | 20:52 | SCxV2.3-BA0004 |
